# Exploration and verification of COVID-19-related hub genes in liver physiological and pathological regeneration

**DOI:** 10.3389/fbioe.2023.1135997

**Published:** 2023-02-23

**Authors:** Jihang Shi, Guangya Li, Xiandun Yuan, Yafei Wang, Ming Gong, Chonghui Li, Xinlan Ge, Shichun Lu

**Affiliations:** ^1^ Medical School of Chinese People’s Liberation Army (PLA), Beijing, China; ^2^ Faculty of Hepato-Pancreato-Biliary Surgery, Chinese PLA General Hospital, Beijing, China; ^3^ Institute of Hepatobiliary Surgery of Chinese PLA, Beijing, China; ^4^ MOE Key Laboratory of Cell Proliferation and Differentiation, College of Life Sciences, Peking-Tsinghua Center for Life Sciences, Peking University, Beijing, China; ^5^ Peking University-Tsinghua University-National Institute of Biological Science Joint Graduate Program, College of Life Science, Peking University, Beijing, China; ^6^ Department of Rheumatology and Immunology, Peking University Third Hospital, Beijing, China

**Keywords:** COVID-19, ALF, liver regeneration, cdc20, Apcin

## Abstract

**Objectives** An acute injury is often accompanied by tissue regeneration. In this process, epithelial cells show a tendency of cell proliferation under the induction of injury stress, inflammatory factors, and other factors, accompanied by a temporary decline of cellular function. Regulating this regenerative process and avoiding chronic injury is a concern of regenerative medicine. The severe coronavirus disease 2019 (COVID-19) has posed a significant threat to people’s health caused by the coronavirus. Acute liver failure (ALF) is a clinical syndrome resulting from rapid liver dysfunction with a fatal outcome. We hope to analyze the two diseases together to find a way for acute failure treatment.

**Methods** COVID-19 dataset (GSE180226) and ALF dataset (GSE38941) were downloaded from the Gene Expression Omnibus (GEO) database, and the “Deseq2” package and “limma” package were used to identify differentially expressed genes (DEGs). Common DEGs were used for hub genes exploration, Protein-Protein Interaction (PPI) network construction, Gene Ontology (GO) functional enrichment, and Kyoto Encyclopedia of Genes and Genomes (KEGG) pathway enrichment. The real-time reverse transcriptase-polymerase chain reaction (RT-qPCR) was used to verify the role of hub genes in liver regeneration during *in vitro* expansion of liver cells and a CCl4-induced ALF mice model.

**Results:** The common gene analysis of the COVID-19 and ALF databases revealed 15 hub genes from 418 common DEGs. These hub genes, including CDC20, were related to cell proliferation and mitosis regulation, reflecting the consistent tissue regeneration change after the injury. Furthermore, hub genes were verified *in vitro* expansion of liver cells and *in vivo* ALF model. On this basis, the potential therapeutic small molecule of ALF was found by targeting the hub gene CDC20.

**Conclusion** We have identified hub genes for epithelial cell regeneration under acute injury conditions and explored a new small molecule Apcin for liver function maintenance and ALF treatment. These findings may provide new approaches and ideas for treating COVID-19 patients with ALF.

## Introduction

COVID-19 is an acute respiratory infectious disease caused by Severe acute Breathing Syndrome Coronavirus 2 (SARS-CoV-2) (Sharma et al., 2021), which may cause damage to multiple systems, including the respiratory and digestive systems (Tregoning et al., 2021). While the pathogenesis of COVID-19 is well understood, the main clinical treatment is still symptomatic. Therefore, it is essential to investigate further the mechanism of epithelial cell regeneration under acute injury, find ways to maintain tissue and organ functions, and avoid chronic impairment (Li et al., 2020a; Delorey et al., 2021; Melms et al., 2021).

ALF is a severe clinical disease usually accompanied by multiple organ failures caused by acute liver damage. It can present as ALF or acute-on-chronic liver failure (ACLF) (Sarin et al., 2019). Overdose of acetaminophen (APAP) is a common cause in most Western countries. In China, acute liver failure caused by HBV infection also has a high incidence (Bunchorntavakul and Reddy, 2018; Dong et al., 2020). Treatment of liver transplantation is often limited by a shortage of liver sources (Olivo et al., 2018). Therefore, other interventions to mitigate liver damage are worth exploring. Despite some similar mechanisms, the prognosis of the ALF and ACLF is significantly different due to changes in liver regeneration properties. Continuous chronic injury destroys the microenvironment of liver cell regeneration, leading to continuous necrosis and apoptosis of the liver and eventually irreversible liver failure (Russo and Parola, 2011; Rastogi et al., 2014; Shubham et al., 2019).

A variety of epithelial cells, including renal tubule, respiratory epithelial, gastric epithelial cell, and liver cell, have certain regeneration capacities under acute injury (Sen et al., 2016; Wilkinson and Hardman, 2020). This regeneration process is the result of coordination among various cell types. Epithelial cells often undergo phenotypic changes, for example, cellular dedifferentiation and activation of regenerative pathways, including non-canonical nuclear factor-kappaB (NF-κB), Janus kinase-signal transducer and activator of transcription (JAK-STAT), phosphatidylinositol 3-kinase/protein kinase B (PI3K/Akt), Wnt, and AP1 (Sen et al., 2016; He and Barron, 2020; Michalopoulos and Bhushan, 2021; Giuliani et al., 2022). In this process, regenerated epithelium helps repair wounds and promote recovery. However, during the regeneration period, the epithelial cell may turn to progenitor-like cells, which affect normal cellular function, increasing the risk of damage in the short term (Yimlamai et al., 2014; Gárate-Rascón et al., 2021). Therefore, it is reasonable to balance motivating cellular regeneration with controlling inflammatory response and maintaining tissue function.

In this study, through bioinformatics analysis of the public databases, we studied the changes in gene expression in COVID-19 and ALF epithelial cells under acute injury and identified hub genes such as *CDC20, CENPF, KIF4, KIF11, NUSAP1, TPX2*, and *PTTG1*. The hub genes and their enrichment pathways helped to clarify the mechanisms of impairment in ALF and COVID-19. Further, the role of hub genes in liver regeneration or ALF was verified by *in vitro* and *in vivo* experiments. The therapeutic effect of an inhibitor for a hub gene *CDC20* on acute liver failure was verified in the ALF mice model, which may provide a new method for treating ALF and COVID-19.

## Methods

### ALF and COVID-19 datasets collection

This article’s related datasets include COVID-19 and ALF from the Gene Expression Omnibus (GEO) database. The COVID-19 dataset (GSE180226) comprises 20 COVID-19 lung samples and three normal lung samples, and the ALF dataset (GSE38941) contains 17 HBV-infected ALF liver samples and ten healthy controls.

### Identification of common DEGs between ALF and COVID-19

The “limma” package in RStudio software (version 4.2.2) was used to identify DEGs with standard | Log2Fold Change | > 1 and | adj. *p*-value | < 0.05 for the COVID-19 dataset (GSE180226). Meanwhile, DEGs with | Log2Fold Change | > 1 and | adj. *p*-value | < 0.05 for the ALF dataset (GSE38941) were also identified. We obtained COVID-19 and ALF common DEGs using the “Venn” package in R software.

### Pathway enrichment analysis of common DEGs

The “clusterProfiler” package in R software (version 4.2.2) was used to perform GO and KEGG enrichment analysis. Other packages, including “dplyr,” “org.Hs.e.g.,.db,” “circlize,” “RColorBrewer,” “ggplot2,” “enrichplot,” “ggpubr,” and “ComplexHeatmap” were used for data annotation and visualization. *p*-value <0.05 was set as the cutoff criterion for common DEGs.

### PPI network analysis based on common DEGs

PPI networks were established by Search Tool for the Retrieval of Interacting Genes (STRING) database based on the combined score >0.9 and visualized by Cytoscape 3.9.1 (version 4.2.2) to reveal the interactions among proteins of common DEGs.

### Verification of hub genes

The expression of the common hub genes in GSE120652 for ALF and GSE139602 for ACLF was detected. GSE120652 contains three acetaminophen-induced ALF samples and three healthy samples, and GSE134431 contains eight ACLF samples and six healthy samples. Wilcoxon test was used to compare the two datasets.

### Human hepatocyte expansion

The cryopreserved human hepatocytes were plated on collagen type I-coated (Rat-tail collagen type I, Gibco) plate at a density of 1 × 10^5^/cm^2^ in a modified liver expansion medium containing William’s E Medium supplemented with 2% B27 (without VA), 1% PS (All from Invitrogen), 50 ng/mL EGF, 20 ng/mL HGF (Both from Peprotech), 200 μM 2-phospho-L-ascorbic acid (pVc), 3 μM CHIR99021, 5 μM SB431542, 5 μM Lysophosphatidic acid (LPA), 0.5 μM Sphingosine-1-phosphate (S1P) (All from MCE), and 1% fetal bovine serum (FBS) (Gibco, US). After seeding for 7–10 days, expanded cells were passaged at a ratio of 1:2 after dissociation with Accutase (eBioscience). The expansion medium was changed every day. Hepatocyte donor information is shown in Table 1.

**TABLE 1:** Donor information on the liver tissues.

Batch id	Age	Gender	Tumor type	pT	pN	cM
6TMY20A30	30	Male	HCC	T1b	N0	M0
6TFY20A67	67	Female	HCC	T2	N0	M0
7TMY21A55	55	Male	HCC	T2	N0	M0

### Real-time reverse transcriptase-polymerase chain reaction (RT-qPCR)

The Direct-zol RNA Kits (ZYMO Research, R2052) were used to isolate total RNA from liver tissues and hepatocytes, followed by reverse transcription using the TransScript First-Strand cDNA Synthesis SuperMix (TransGen, AT311). The KAPA SYBR^®^ FAST qPCR Kit (KAPA Biosystems, KK4601) was used to perform relative gene expression on a CFX384 Touch Real-Time PCR Detection System (Bio-Rad). The housekeeping genes (RRN18S) were used as an internal reference to analyze RT-qPCR results using the 2^(−ΔΔCt) method. All the primer sequences for RT-qPCR are provided in Table 2.

**TABLE 2:** Primer sequences for RT-qPCR in the study.

Gene	Forward primer (5′ to 3′)	Reverse primer (5′ to 3′)
**Mus musculus genes**		
Rn18s	CGC​GGT​TCT​ATT​TTG​TTG​GT	AGT​CGG​CAT​CGT​TTA​TGG​TC
*Alb*	TTA​GTG​CAG​GAA​GTA​ACA​GAC	AAG​AGT​GTG​AAG​GGA​TTT​GTC
*Cyp7a1*	GGA​ATA​AGG​AGA​AGG​AAA​GTA​GGT	TCC​GAA​CTT​CAG​AGC​ACA​G
*Cyp3a11*	ACT​CAA​GGA​GAT​GTT​CCC​TG	TTG​CCT​TTC​TTT​GCC​TTC​TG
*Epcam*	GTT​GTC​CTG​GTT​ATA​TCT​ACA​AGG	TCT​CTG​TGG​ATC​TCA​CCC​A
*Afp*	CTT​AGC​ATA​GCT​ACC​ATC​ACC	TCT​TCA​TTG​CAG​CCA​ACA​C
*Krt19*	ATT​GAC​AAC​TCC​AAG​ATT​GTC​C	TTC​TGT​CTC​AAA​CTT​GGT​TCT​G
*CDC20-F*	ACT​GAA​AGT​ACT​GTA​CAG​TCA​G	GAT​TCA​GGT​AGT​AGT​CAT​TCC​G
*Cenpf*	AAA​CCA​GAC​TTA​CCC​AGG​A	CTG​ATG​TGA​CCT​TCT​CCA​G
*Cep55*	CTC​TAC​GAT​TCT​CTG​TTA​AAG​CAC	TAG​AGT​ACA​TGC​CTG​CAT​CTG
*Kif4*	ATG​ACA​GAG​AAG​CAG​CTG​G	CCT​AAG​CTC​TTC​CTC​CTG​AC
*Kif11*	GGA​GCT​CAG​TAA​GGC​TAC​AG	GTC​TTG​GTT​TGC​AGG​TCA​G
*Ncapg*	GCT​TGT​TGA​TCT​GAC​AAG​AC	TCA​TGT​ACT​GTT​AAG​GCC​TG
*Nusap1*	AAC​GAA​CGT​GTA​AGC​AGA​G	CTT​CCA​TCG​TTC​TTC​CCT​G
*Pttg1*	GAT​AAG​GAT​AAT​GAA​GAA​CCC​GG	ATC​TAA​GGC​CTT​GAC​ACC​A
*Tpx2*	CCC​ATC​AAA​TGA​TTG​TTC​TTC​C	ATA​GAT​CTT​CTC​TGA​GGC​TGG
*Ube2c*	CTG​ATG​TGA​CCT​TCT​CCA​G	TGT​TGG​GTT​CTC​CTA​GCA​G
**Homo sapiens genes**		
*18S*	GTA​ACC​CGT​TGA​ACC​CCA​TT	CCA​TCC​AAT​CGG​TAG​TAG​CG
*AFP*	CCC​GAA​CTT​TCC​AAG​CCA​TA	TAC​ATG​GGC​CAC​ATC​CAG​G
*ALB*	GCA​CAG​AAT​CCT​TGG​TGA​ACA​G	ATG​GAA​GGT​GAA​TGT​TTC​AGC​A
*EPCAM*	TGA​TCC​TGA​CTG​CGA​TGA​GAG	CTT​GTC​TGT​TCT​TCT​GAC​CCC
*KRT19*	TGA​GCA​GGT​CCG​AGG​TTA​CTG	CAG​TGT​GTC​TTC​CAA​GGC​AGC
*CYP2C9*	GCC​ACA​TGC​CCT​ACA​CAG​ATG	TAA​TGT​CAC​AGG​TCA​CTG​CAT​GG
*CYP1A2*	CTT​CGT​AAA​CCA​GTG​GCA​GG	AGG​GCT​TGT​TAA​TGG​CAG​TG
*CPS1*	AAT​GAG​GTG​GGC​TTA​AAG​CAA​G	AGT​TCC​ACT​CCA​CAG​TTC​AGA
*NAGS*	CAG​CAG​GGC​GTA​TCC​AGT​C	GTT​GTG​TCG​AAG​CGC​GTC​TA
*CYP3A4*	GGT​GGT​GAA​TGA​AAC​GCT​CAG	ACC​CCT​TTG​GGA​ATG​AAC​ATC
*CDC20*	GCA​CAG​TTC​GCG​TTC​GAG​A	CTG​GAT​TTG​CCA​GGA​GTT​CGG
*CENPF*	CTC​TCC​CGT​CAA​CAG​CGT​TC	GTT​GTG​CAT​ATT​CTT​GGC​TTG​C
*CEP55*	AGT​AAG​TGG​GGA​TCG​AAG​CCT	CTC​AAG​GAC​TCG​AAT​TTT​CTC​CA
*KIF4A*	TAC​TGC​GGT​GGA​GCA​AGA​AG	CAT​CTG​CGC​TTG​ACG​GAG​AG
*KIF11*	TCC​CTT​GGC​TGG​TAT​AAT​TCC​A	GTT​ACG​GGG​ATC​ATC​AAA​CAT​CT
*NCAPG*	GAG​GCT​GCT​GTC​GAT​TAA​GGA	AAC​TGT​CTT​ATC​ATC​CAT​CGT​GC
*NUSAP1*	AGC​CCA​TCA​ATA​AGG​GAG​GG	ACC​TGA​CAC​CCG​TTT​TAG​CTG
*PTTG1*	ACC​CGT​GTG​GTT​GCT​AAG​G	ACG​TGG​TGT​TGA​AAC​TTG​AGA​T
*TPX2*	ATG​GAA​CTG​GAG​GGC​TTT​TTC	TGT​TGT​CAA​CTG​GTT​TCA​AAG​GT
*UBE2C*	GAC​CTG​AGG​TAT​AAG​CTC​TCG​C	TTA​CCC​TGG​GTG​TCC​ACG​TT

### The acute liver failure model

The animal experiments were conducted under a protocol (2019-X15-03) that the ethics committee approved by the Laboratory Animals Center of Chinese PLA General Hospital (Beijing). Twenty-one male C57BL/6J mice (weight 30 g, aged 8–10 weeks) were purchased from Sipeifu (Beijing, China) and randomly divided into three groups to obtain liver tissue for molecular and histological studies (*n* = 7/group): mice with corn oil only without CCl4 injury (NC), mice with corn oil and CCl4 injury (CCl4), and mice with Apcin and CCl4 injury (Apcin). Before the CCl4 injection, the Apcin mice group was intraperitoneally injected with Apcin (30 mg/kg) dissolved in corn oil. Eight hours later, the CCl4 and Apcin mice were given CCl4 2.9 mL/kg dissolved in corn oil (Solarbio, C7030) by intraperitoneal injection. The mice in the NC group were injected with corn oil as a control. After 24 h of CCl4 treatment, all mice were sacrificed to obtain serum and liver tissues. Another 21 mice were randomly divided into three groups (*n* = 7/groups) to obtain the survival rate of ALF, as described above. Seven days after injection, all mice were sacrificed, and the survival rate can be further analyzed.

### Serum biochemical examination

0.8 mL serum was incubated for 30 min and then centrifuged at 3,000 rpm for 5 min to get the supernatant to collect the mouse blood sample. The automatic chemistry analyzer was used to measure the aspartate aminotransferase (AST) and alanine aminotransferase (ALT), with each biochemical parameter being repeated four times.

### Immunofluorescence staining

Hepatocytes on the glass bottom plate (Cellvis, P24-1.5H-N) were fixed with 4% paraformaldehyde in PBS at room temperature for 20 min. Then the cells permeabilized in 0.25% Triton X-100 and blocked with 2.5% normal donkey serum (Jackson ImmunoResearch Laboratories, 017-000-001) in PBS. Next, the hepatocytes were incubated with primary antibodies at room temperature for 1 h, including Anti-ALB (ab243894, Abcame, 1:200), Anti-CDC20 (sc-5296, Abcame, 1:200), and Anti-CK19(ab181557, Abcame, 1:250). Hepatocytes were incubated with the secondary antibodies, including DyLight^®^ 650 Donkey anti-rabbit (ab96894, Abcame, 1:200) and DyLight^®^ 650 Donkey anti-mouse (ab96878, Abcame, 1:200) for 1 h at room temperature. Finally, the samples were stained with 4′,6-diamidino-2-phenylindole (DAPI) (Invitrogen, D1306) for 5 min and observed and photographed under an inverted fluorescent microscope using Nikon Imaging System at the same exposure.

### Immunohistochemistry and hematoxylin-eosin (H&E) staining

After paraffin-embedded, the liver tissue was cut into sections of 4 μm thickness. Then the slides immunohistochemistry for the TUNEL staining was performed using the TUNEL Assay Kit-HRP-DAB (Abcam, ab206386). The H&E staining was performed using a regular protocol. All the results were randomly taken at the same exposure.

### Statistical analysis

Graph Pad 8.0 was used for statistical analysis. All the data were the means ± standard deviation (SD). Differences with *p* < 0.05 was considered statistically significant. Student’s *t*-test and Wilcox were used to analyze differences between two or multiple groups. Kaplan-Meier analysis was used for the overall survival.

## Results

### Acquirement of the common DEGs between ALF and COVID-19

**FIGURE 1:**
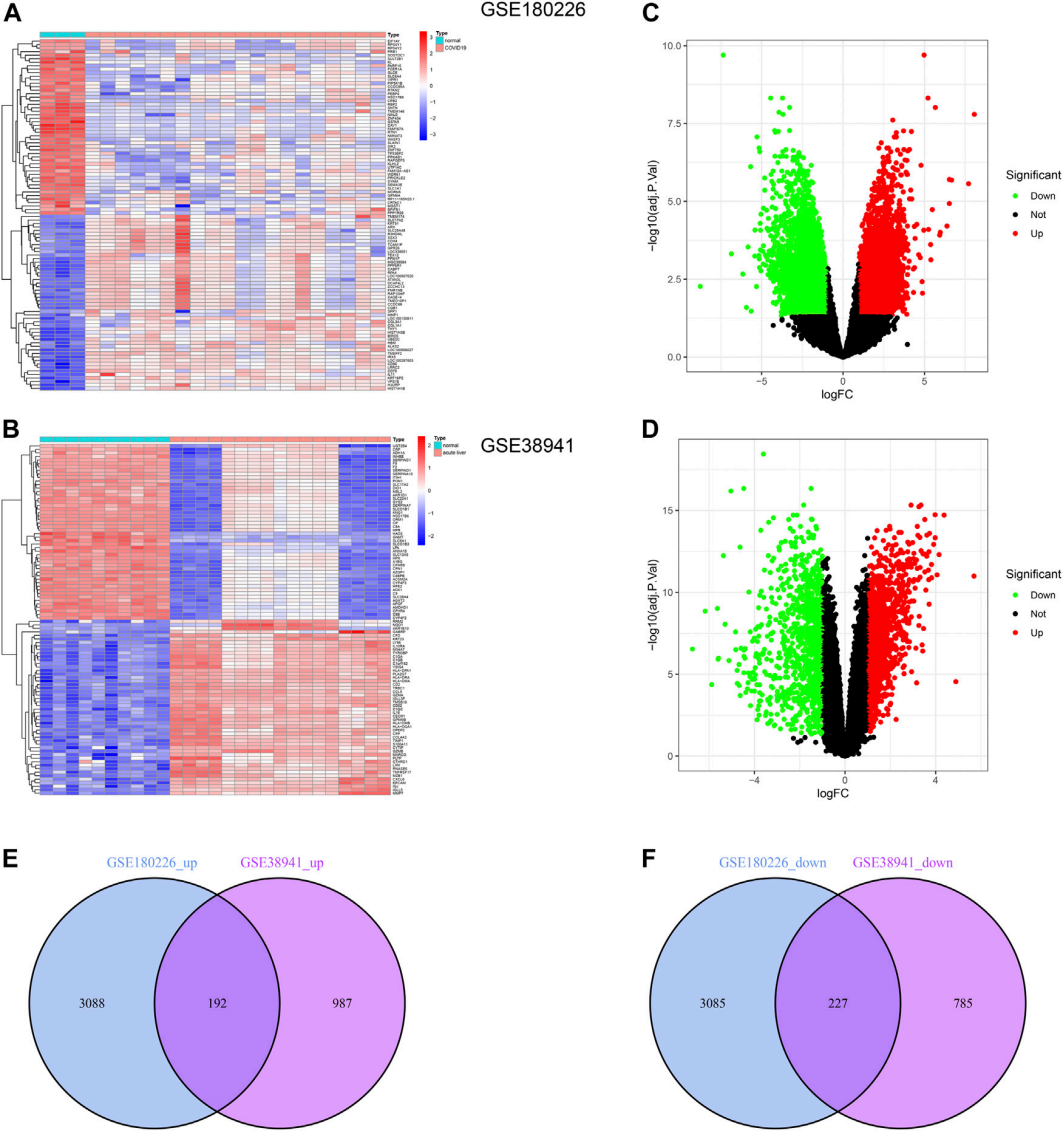
Identification of common DEGs between COVID-19 and ALF. **(A)** Heatmap of the DEGs in COVID-19 patients compared with healthy controls. **(B)** Heatmap of the DEGs in ALF patients compared with healthy controls. **(C)** Volcano plot of DEGs in COVID-19 patients, in which green represents downregulated DEGs, red represents upregulated DEGs, and black represents no difference. **(D)** Volcano plot of DEGs in ALF. And the colors represent the same sample as B. **(E)** Venn diagram showing the upregulated DEGs between COVID-19 and ALF patients. **(F)** Venn diagram showing the downregulated DEGs between COVID-19 and ALF patients.

To explore the common changes of epithelial cells under acute injury to find potential targets for injury recovery and tissue regeneration, we downloaded the transcriptome data of COVID-19 and ALF from the GEO database. We identified 6,592 and 2,191 DEGs by comparing them with healthy data (Figures 1A,B). Further, the volcano plots demonstrated DEGs of ALF and COVID-19, respectively (Figures 1C,D). One hundred ninety-two co-upregulated genes and 227 co-downregulated genes were shown in Venn diagrams between ALF and COVID-19 datasets (Figures 1E, F and Supplementary Material S1). These results indicated common mechanisms and genetic expression trends among different tissues under acute injury.

### GO and KEGG analysis of common DEGs

**FIGURE 2:**
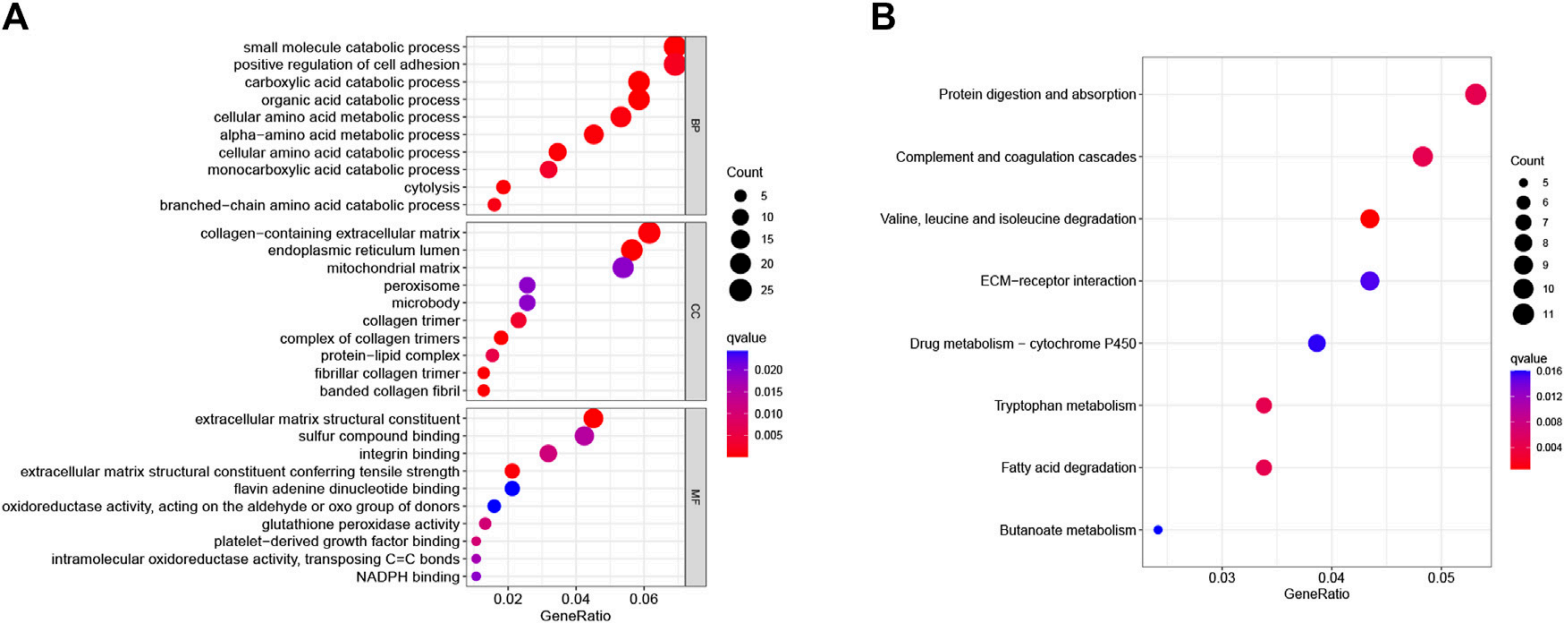
GO and KEGG pathway analyses of common DEGs. **(A)** The top ten GO functional enrichment analyses of the common DEGs in biological process (BP), cell composition (CC), and molecular function (MF) groups, respectively. The bubble size in the dotplot shows the first five terms, and the size represents p-adjusted values for these terms. **(B)** The top eight significant KEGG signal pathways of the common DEGs.

GO and KEGG enrichment analyses were used further to analyze the role and function of common DEGs. GO enrichment analysis included three categories: biological process (BP), cell composition (CC), and molecular function (MF). The common DEGs were mainly enriched in amino acid metabolic processes or metabolic processes of other substances (BP), the complex of collagen trimers, endoplasmic reticulum lumen, and fibrillar collagen trimer (CC), and extracellular matrix reconstruction and antioxidant stress response (MF) (Figure 2A). The KEGG enrichment revealed that the common DEGs mainly enriched in protein digestion and absorption, complement and coagulation cascades, and drug metabolism pathways (Figure 2B).

### PPI network construction and hub genes extraction

**FIGURE 3:**
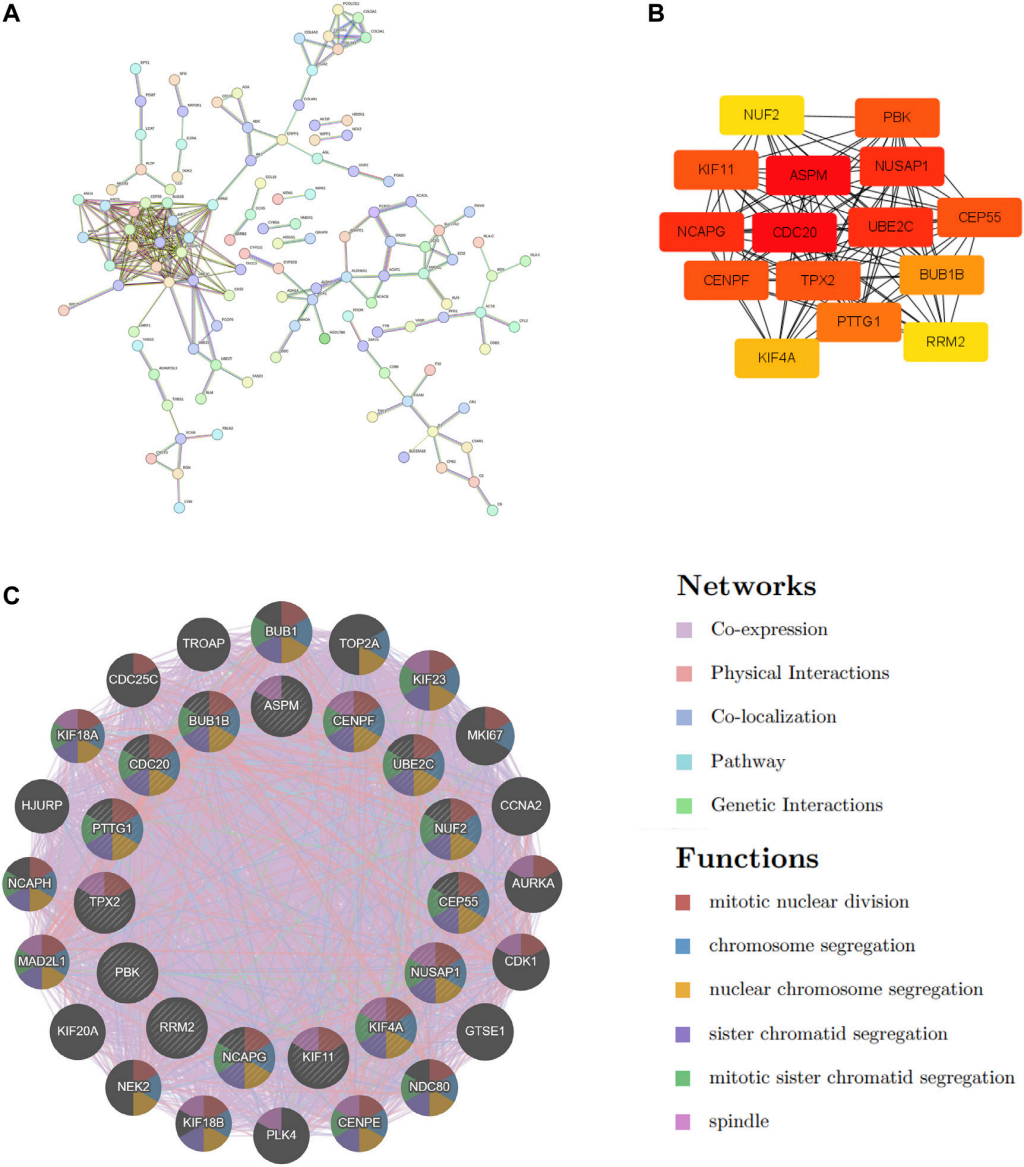
PPI Network Construction and hub genes extraction. **(A)** COVID-19 and ALF common DEGs in the PPI network constructed by STRING. **(B)** The top 15 hub genes and their interactions with each other. **(C)** The gene–gene interaction network was visualized by the GeneMANIA database. The top 20 mostly related neighboring genes are shown in the outer circle of hub genes.

**FIGURE 4:**
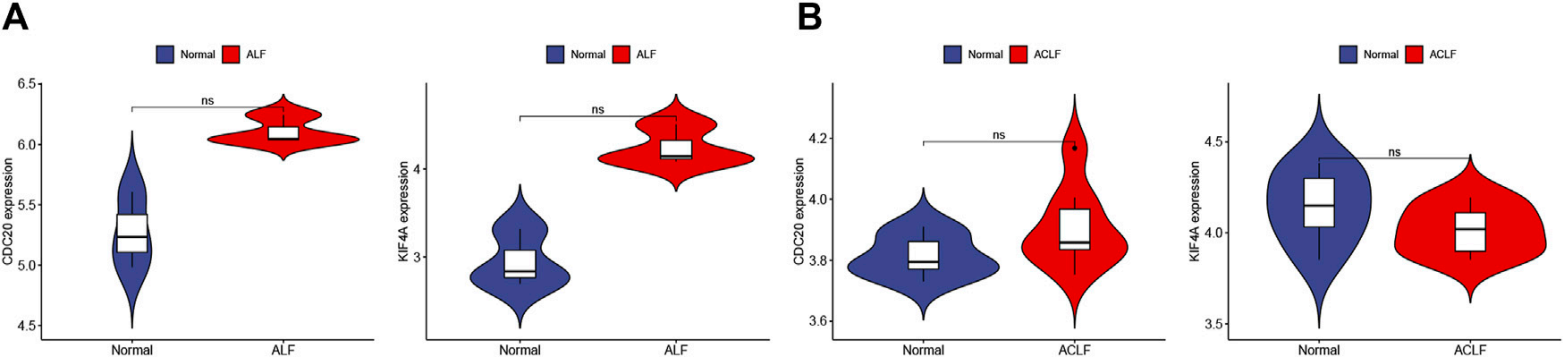
Validation of hub genes in ALF and ACLF by Violin plots. **(A)** The expression of representative hub genes between livers of ALF patients with normal livers was compared. **(B)** The expression of representative hub genes in ACLF and normal livers was compared. Eight ACLF and six control healthy livers were obtained from GSE139602. Data were statistically analyzed using the Wilcoxon test (ns indicates no significant difference).

STRING was used to construct the PPI network to explore gene interactions further and establish a hub gene network, in which 81 nodes and 250 edges were included (Figure 3A). CytoHubba was used to identify the top 15 genes in Cytoscape based on the degree method scores in the PPI network, including *BUB1B, CDC20, ASPM, NUSAP1, CEP55, UBE2C, KIF11, RRM2, PBK, PTTG1, NCAPG, TPX2, KIF4A, NUF2*, and *CENPF* (Figures 3B,C). We believe these targets may reflect the change of epithelial cells in the state of acute injury. Validation was performed for ALF and ACLF from GEO, and the results showed that these hub genes were upregulated in ALF compared to normal tissues, although without significant differences (Figures 4A, B). However, the trend is not evident in ACLF (Figures 4A, B). These results indicate that chronic injury weakens the regenerative ability of epithelial cells, consistent with current theoretical studies, suggesting the role of hub genes in acute injury from the side.

### Functional enrichment analysis of hub genes

**FIGURE 5:**
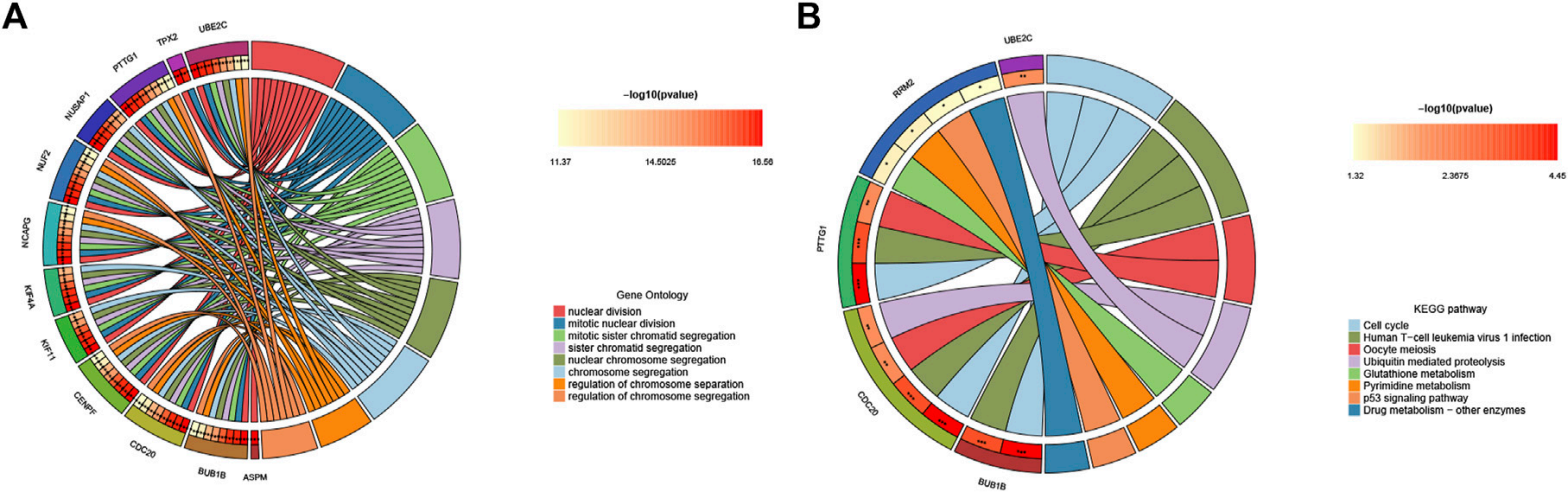
Functional enrichment analysis of hub genes. **(A)** Circle diagram visualization of GO terms, and **(B)** top eight significant KEGG pathways. For each graph, the right semicircle represents significant pathways. Different color modules represent different pathways. And the left semicircle represents the genes enriched in the same color pathways.

To further explore hub genes’ biological effects on tissue injury, GO and KEGG analyses were conducted again on the 15 hub genes. Interestingly, the enrichment status of hub genes was apparent. In GO enrichment, the BP category, CC category, and MF category all showed functional enrichment related to cell proliferation, mitosis, and spindle formation. KEGG enrichment analysis demonstrated pathways associated with cell proliferation and viral infection (e.g., Cell cycle, Human T−cell leukemia virus one infection, and Oocyte meiosis) (Figures 5A, B). These results indicate that tissue regeneration is the most apparent phenomenon under the stress of acute failure.

### *In vitro* and *in vivo* models of liver regeneration

**FIGURE 6:**
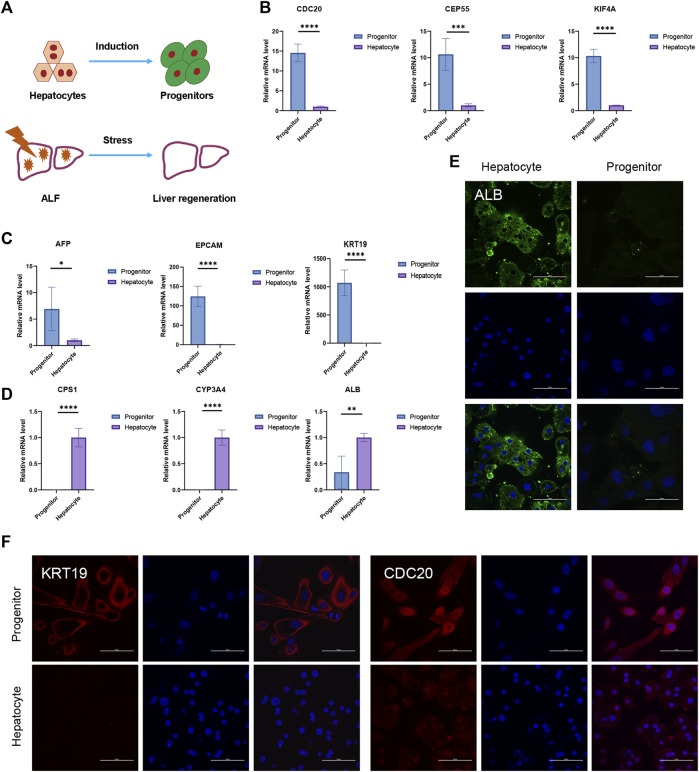
*In vitro* liver regeneration under physiological state. **(A)** Damage and regeneration pattern diagram. The process of hepatocytes turning into progenitors represents *in vitro* regeneration model. Liver regeneration occurs during ALF and represents *in vivo* regeneration model. **(B–D)** The relative expression of hub genes **(B)**, progenitor genes **(C)**, and liver functional genes **(D)** between hepatocytes and progenitors. *N* = 4. Data were statistically analyzed using Student’s *t*-test, and values were presented as mean ± SD. *indicates significant difference at *p* < 0.05, ** indicates significant difference at *p* < 0.01, *** indicates significant difference at *p* < 0.001, **** indicates significant difference at *p* < 0.0001. **(E–F)** Representative immunofluorescence staining images of ALB **(E)**, KRT19, and CDC20 **(F)** (Scale bars = 100 µm).

**FIGURE 7:**
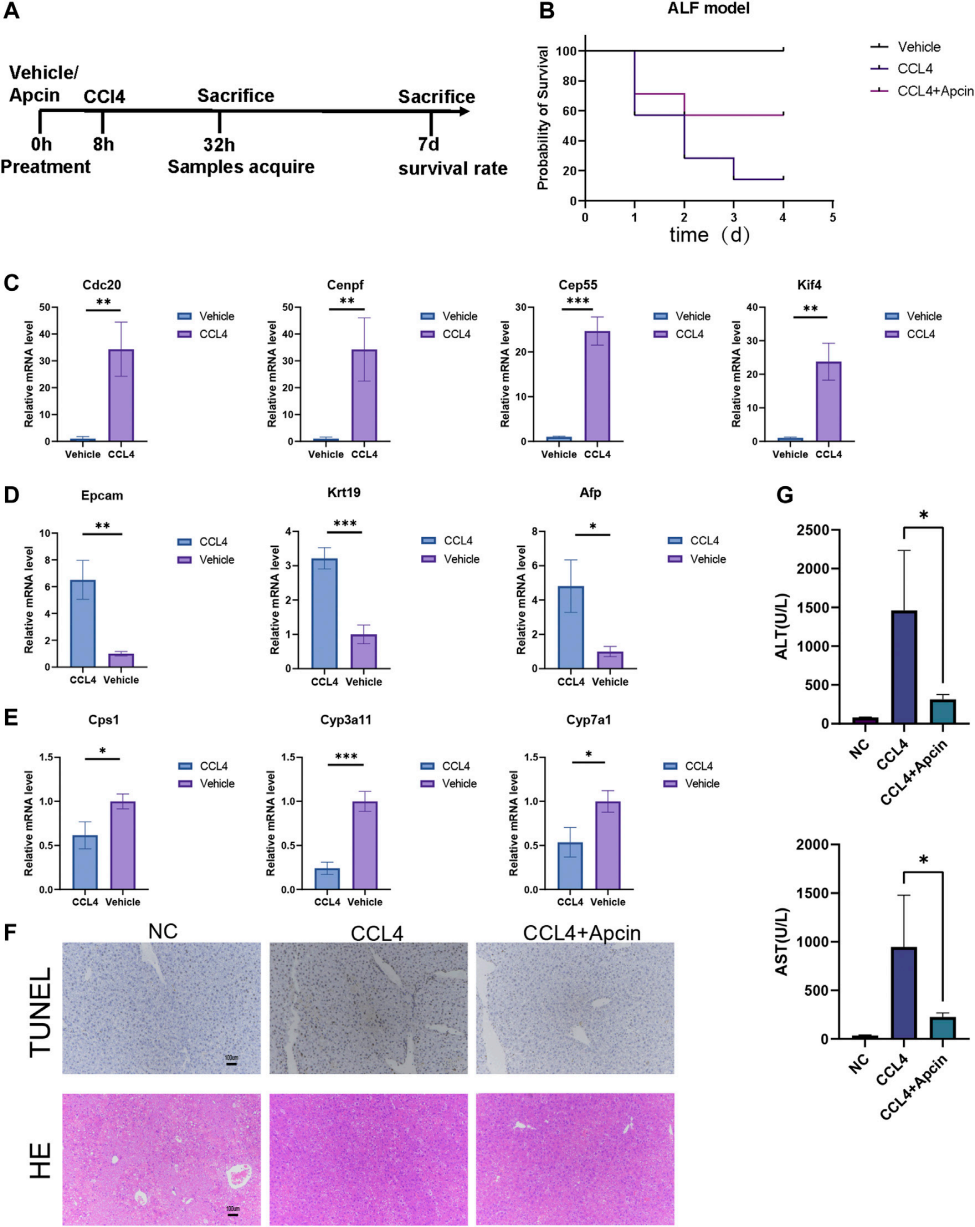
CDC20 inhibitor Apcin alleviates ALF. **(A)** The flow diagram demonstrates the ALF model construction and treatment. **(B)** The survival rate of ALF mice with or without Apcin pretreatment. *N* = 7. **(C–E)** The relative expression of hub genes **(C)**, progenitor genes **(D)**, and functional genes **(E)** between ALF and vehicle mice. *N* = 3. **(F)** Representative TUNEL (upper panels) and H&E (lower panels) staining of liver tissues harvested at 24 h post CCL4 induction with or without Apcin pretreatment. *N* = 3 (Scale bars = 100 µm). **(G)** The AST and ALT levels in NC, CCl4 with or without Apcin mice groups. *N* = 4. Data were statistically analyzed using Student’s *t*-test, and values were presented as mean ± SD. *indicates significant difference at *p* < 0.05, ** indicates significant difference at *p* < 0.01, *** indicates significant difference at *p* < 0.001.

In the *in vitro* hepatocyte regeneration model, serum and cytokines simulating the injury environment were used to induce hepatocyte proliferation (Figure 6A). Hepatocytes entered the progenitor cell state with the upregulation of hub genes such as *CDC20, CENPF, CEP55, KIF4A, KIF11, NCAPG, NUSAP1, PTTG1, TPX2*, and *UBE2C* (Figure 6B, Supplementary Material S2A). Compared to the primary hepatocytes, progenitor markers such as *EPCAM*, *AFP*, and *CK19* expressed much higher in liver progenitors while liver function genes including cytochrome P450 (CYP) enzymes and urea metabolism genes downregulated (Figures 6C, D, Supplementary Material S2A). Immunofluorescence staining confirmed the changes in cellular identity and function (Figures 6E, F). These results demonstrated the relationship between hub genes and liver regeneration. The *in vivo* experiment schematic is represented in Figure 7A. Similarly, in the CCL4-induced ALF model to monitor liver injury and regeneration, we also observed significantly upregulated hub genes in the ALF liver than healthy control (Figure 7C, Supplementary Material S2B). In addition, progenitor genes were upregulated, while CYP enzymes and other functional genes were down (Figures 7D, E, Supplementary Material S2B). These results reflect the consistent changes in response to stress stimulation and following regeneration patterns in liver cells.

### The inhibitor Apcin for bub gene CDC20 alleviates ALF

During ALF, extensive hepatocytes undergo dedifferentiation, and proliferation may influence liver function. Therefore, we wonder if maintaining the identity of hepatocytes and moderate inhibition of proliferation will contribute to the survival of ALF. CDC20 inhibitor Apcin has been reported to inhibit cancer cell proliferation by competitively inhibiting anaphase-promoting complex/Cyclosome (APC/C)-dependent ubiquitination. It was found that Apcin pretreatment can attenuate liver failure and improve the survival rate in a mouse model (Figure 7B). Liver damage and inflammatory response were relieved by Tunnel and H&E staining (Figure 7F). Serum of mice was collected for biochemical detection, including alanine aminotransferase (ALT) and aspartate transaminase AST (Figure 7G). These data illustrate the positive effect of Apcin on ALF, which may also help with the treatment of COVID-19.

In conclusion, these results indicate that regardless of COVID-19, ALF, or other acute failures, the epithelium entered the cell proliferation stage induced by injury, and this regeneration contributed to repairing and recovery in the long run. However, short-term loss of function may aggravate the general condition. Therefore, proper regulation of the balance between cell proliferation and function maintenance may be beneficial for patients to recover from acute failure. The hub genes identified in this paper and their corresponding targets, such as CDC20 inhibitor-Apcin, may be a new possible approach for ALF and COVID-19 treatment.

## Discussion

In the study of tissue regeneration after acute injury, we analyzed the database of ALF and COVID-19 by bioinformatics method and found common hub genes, including *CDC20, CENPF, KIF4, KIF11, NUSAP1, TPX2,* and *PTTG1,* which were related to mitosis and cell cycle regulation. They were highly expressed in patients with ALF and COVID-19, reflecting epithelial tissue’s regeneration and repair response to acute injury. We confirmed that these hub genes showed significantly enhanced expression during *in vitro* hepatocyte regeneration and mouse models with ALF. Therefore, we conclude that these genes may play an essential role in epithelial damage and regeneration. Further *in vivo* studies confirmed that targeting CDC20 to maintain hepatocyte identity and liver function is beneficial for mice to survive ALF. Perhaps it can be further extended to the treatment of COVID-19.

The lungs are the primary site of immunopathology induced by SARS-CoV-2, and angiotensin-converting enzyme 2 (ACE2) are receptors for the virus to enter host cells (Mulay et al., 2021). Complex lung regeneration and repair programs exist to replenish damaged cell populations. In addition to clinical treatments being applied, it is necessary to promote lung regeneration for COVID-19 patients (Zhao et al., 2022).

For treating ALF, the current research mainly focuses on two aspects. The liver is the organ with the strongest regenerative capacity in the human body, and after a temporary injury, the liver can still regenerate and recover. Therefore, inhibiting inflammatory response, eliminating inhibition signal, and promoting hepatocyte regeneration are effective means for treating ALF (Rutherford and Chung, 2008; Larsen, 2019). However, studies have found that liver cells cannot maintain their properties under ALF conditions and undergo dedifferentiation and proliferation, which may lead to impaired liver function, increased blood ammonia and bilirubin, and aggravated inflammation (Ko et al., 2020). Therefore, maintaining the function of the liver is also an essential means of ALF treatment.

In conclusion, it is of great significance to find the hub genes in the state of acute injury for treating COVID-19 infection and ALF.

CDC20, part of the anaphase-promoting complex/cyclosome (APC/C), has been recognized as necessary in cell cycle regulation. APC/CCDC20 promotes the ubiquitination and degradation of critical proteins to influence various cellular processes (Wang et al., 2015). *CDC20* and *KIF11* were listed as hub genes for COVID-19 treatment and novel therapeutic targets against SARS-CoV-2 infections (Auwul et al., 2021). Moreover, they were identified as biomarkers of HIV-infected COVID-19 patients (Yan et al., 2022). Based on the study of myocardial injury and myocardial regeneration differences among different ages, *CDC20* was identified as the critical factor of myocardial regeneration (Locatelli et al., 2020). In addition, the promoting effect of *CDC20* was found in muscle regeneration, corneal regeneration, and liver regeneration studies (Kuwata et al., 2011; Song et al., 2022). *CDC20* has been reported as a promoter in human tumorigenesis. Overexpression of *CDC20* is associated with clinicopathologic features of various human tumors. These tumors include hepatocellular carcinoma (HCC), non-small cell lung cancer (NSCLC), pancreatic tumor, and colorectal cancer (Li et al., 2017; Li et al., 2020b; Dai et al., 2021; Gong et al., 2021). All these demonstrated the role of *CDC20* in normal tissue regeneration and tumorigenesis. However, whether *CDC20* works in acute liver failure remains unclear. This study innovatively adopted the CDC20 inhibitor Apcin for treating ALF, and a significant therapeutic effect was achieved in the mouse model of ALF (Ramavath et al., 2021).

*PTTG1, CENPF, KIF4, KIF11, NUSAP1*, and *TPX2* have also been implicated in many biological functions, such as DNA repair, organ development, and regulation of mitosis (Försti et al., 2016). *CENPF* was viewed as a prognostic indicator of lung cancer patients with COVID-19 infection (Cury et al., 2023). *TPX2* and *KIF11* were upregulated in peripheral blood mononuclear cells and contributed to diagnosing and managing COVID-19 patients (Hasan et al., 2022). It is worth noting that KIF4 and KIF11, molecular motor proteins, have been identified as powerful targets contributing to the regeneration of injured axons. *CENPF*, *NUSAP1*, *UBE2C*, and *CDC20* are identified as markers of transit-amplifying cells (TACs) by scRNA-seq and are related to proliferation stages. They are also considered independent prognostic markers for various tumors and play an essential role in tumor progression, metastasis, tumor stem cell formation, and epithelial-mesenchymal transition (Parte et al., 2019; Guo et al., 2020).

In conclusion, these hub genes are essential in tumorigenesis, acute injury, and tissue regeneration and may become potential targets for treating these diseases.

The study had several limitations. First, we have emphasized the role of hub genes in tissue injury and regeneration but lack further exploration of the mechanisms. Second, the therapeutic effect of Apcin for COVID-19 is mainly based on theoretical speculation and lacks some experimental verification. Third but not negligible, in current study, we compared sequencing data between liver and lung tissues, which may have biased the experimental results. Therefore, acquiring and analyzing liver tissue samples of ALF induced by different etiologies including COVID-19 infection may be essential for the next study.

## Conclusion

In this paper, we have identified hub genes that worked in different epithelial tissue regeneration after injury. The results were verified by hepatocyte expansion and ALF model under physiological conditions. In addition, the treatment of ALF by Apcin, a CDC20 inhibitor, was reported for the first time. This discovery could help develop new means or drugs to treat COVID-19 and ALF.

## Data Availability

Publicly available datasets were analyzed in this study. This data can be found here: https://www.ncbi.nlm.nih.gov/geo/query/acc.cgi?acc=GSE180226, https://www.ncbi.nlm.nih.gov/geo/query/acc.cgi?acc=GSE38941, https://www.ncbi.nlm.nih.gov/geo/query/acc.cgi?acc = GSE120652, https://www.ncbi.nlm.nih.gov/geo/query/acc.cgi?acc = GSE139602.
