# Supplementary material for: Exploration and verification of COVID-19-related hub genes in liver physiological and pathological regeneration
Source: Front Bioeng Biotechnol. 2023 Feb 23;11:1135997. doi: 10.3389/fbioe.2023.1135997 (PMC9997844; doi:10.3389/fbioe.2023.1135997)
Supplement: Supplementary file 1 [file Table1.DOCX]

**One hundred ninety-two co-upregulated genes and 227 co-downregulated genes between ALF and COVID-19 datasets**

| **ID** | **Type** | **GES38941_adj. P value** | **GSE180226_adj. P value** | **ID** | **Type** | **GES38941_adj. P value** | **GSE180226_adj. P value** |
| --- | --- | --- | --- | --- | --- | --- | --- |
| ***GSTA1*** | **down** | **3.56E-02** | **1.36E-06** | ***CD86*** | **up** | **1.32E-12** | **9.61E-09** |
| ***CA2*** | **down** | **4.68E-09** | **2.67E-06** | ***UBE2C*** | **up** | **2.03E-05** | **5.67E-08** |
| ***HYAL1*** | **down** | **2.09E-05** | **3.12E-06** | ***NUSAP1*** | **up** | **2.76E-05** | **8.50E-08** |
| ***PGM1*** | **down** | **2.76E-08** | **4.57E-06** | ***ORC6*** | **up** | **5.56E-07** | **1.94E-07** |
| ***CLU*** | **down** | **4.91E-08** | **1.67E-05** | ***EMILIN1*** | **up** | **1.20E-06** | **3.83E-07** |
| ***EMP2*** | **down** | **3.65E-10** | **2.72E-05** | ***TACC3*** | **up** | **2.07E-10** | **3.98E-07** |
| ***NDUFV2*** | **down** | **3.42E-05** | **2.80E-05** | ***BGN*** | **up** | **1.04E-09** | **5.20E-07** |
| ***GRHPR*** | **down** | **3.85E-06** | **3.50E-05** | ***THY1*** | **up** | **2.39E-07** | **1.36E-06** |
| ***PAPSS2*** | **down** | **3.90E-07** | **3.93E-05** | ***E2F3*** | **up** | **5.67E-10** | **3.54E-06** |
| ***LNX2*** | **down** | **1.74E-08** | **4.93E-05** | ***NCAPG*** | **up** | **3.49E-06** | **4.95E-06** |
| ***C3*** | **down** | **3.86E-06** | **5.00E-05** | ***C4orf48*** | **up** | **9.68E-12** | **9.42E-06** |
| ***FRY*** | **down** | **5.89E-08** | **5.83E-05** | ***ANLN*** | **up** | **2.87E-06** | **1.22E-05** |
| ***ACAT1*** | **down** | **3.34E-15** | **6.26E-05** | ***KRT8*** | **up** | **1.17E-08** | **1.69E-05** |
| ***CLRN3*** | **down** | **8.46E-04** | **6.54E-05** | ***SFN*** | **up** | **2.65E-06** | **1.79E-05** |
| ***RARRES2*** | **down** | **1.03E-07** | **6.72E-05** | ***ASPM*** | **up** | **1.97E-05** | **4.30E-05** |
| ***TTPA*** | **down** | **8.39E-10** | **7.01E-05** | ***NXN*** | **up** | **8.91E-05** | **4.84E-05** |
| ***NR3C2*** | **down** | **2.20E-05** | **7.55E-05** | ***CPNE5*** | **up** | **6.01E-04** | **4.93E-05** |
| ***SSTR1*** | **down** | **1.31E-08** | **7.85E-05** | ***SPATS2*** | **up** | **1.23E-10** | **6.09E-05** |
| ***EPB41L5*** | **down** | **6.16E-13** | **8.34E-05** | ***PAPLN*** | **up** | **6.51E-08** | **6.42E-05** |
| ***CFL2*** | **down** | **2.12E-10** | **8.49E-05** | ***TCF19*** | **up** | **5.77E-07** | **6.96E-05** |
| ***CYB5A*** | **down** | **2.37E-05** | **8.55E-05** | ***COL6A3*** | **up** | **2.76E-07** | **8.21E-05** |
| ***C3orf80*** | **down** | **4.22E-06** | **8.62E-05** | ***IL21R*** | **up** | **3.59E-08** | **8.93E-05** |
| ***SEC63*** | **down** | **1.01E-11** | **9.99E-05** | ***6-Sep*** | **up** | **7.73E-09** | **9.27E-05** |
| ***ARHGEF26*** | **down** | **4.04E-10** | **1.13E-04** | ***TRNP1*** | **up** | **1.19E-09** | **1.00E-04** |
| ***HPGD*** | **down** | **6.73E-09** | **1.23E-04** | ***PRR11*** | **up** | **1.76E-12** | **1.01E-04** |
| ***ADI1*** | **down** | **3.09E-09** | **1.29E-04** | ***FAP*** | **up** | **1.81E-07** | **1.05E-04** |
| ***ADHFE1*** | **down** | **4.49E-11** | **1.45E-04** | ***FAM60A*** | **up** | **9.14E-11** | **1.35E-04** |
| ***ATRN*** | **down** | **9.95E-09** | **1.58E-04** | ***RSPO3*** | **up** | **1.15E-04** | **1.62E-04** |
| ***EXPH5*** | **down** | **2.06E-11** | **2.02E-04** | ***CTSC*** | **up** | **2.63E-09** | **1.65E-04** |
| ***ALDH6A1*** | **down** | **1.92E-12** | **2.12E-04** | ***KCNAB2*** | **up** | **1.56E-10** | **1.92E-04** |
| ***PID1*** | **down** | **9.31E-15** | **2.65E-04** | ***ISLR*** | **up** | **2.86E-03** | **1.93E-04** |
| ***SLC1A1*** | **down** | **9.31E-15** | **2.67E-04** | ***DYSF*** | **up** | **1.19E-09** | **2.06E-04** |
| ***IMPA2*** | **down** | **2.03E-06** | **2.86E-04** | ***VASP*** | **up** | **5.37E-09** | **2.12E-04** |
| ***RPRD1B*** | **down** | **8.31E-11** | **3.12E-04** | ***PTTG1*** | **up** | **1.20E-08** | **2.32E-04** |
| ***ABHD6*** | **down** | **3.09E-07** | **3.17E-04** | ***ADA*** | **up** | **2.33E-10** | **2.33E-04** |
| ***PEMT*** | **down** | **2.26E-04** | **3.30E-04** | ***CECR1*** | **up** | **4.37E-13** | **2.38E-04** |
| ***PCYOX1*** | **down** | **3.37E-08** | **3.55E-04** | ***FER1L4*** | **up** | **2.35E-12** | **2.51E-04** |
| ***WASF3*** | **down** | **1.31E-06** | **3.82E-04** | ***PHF19*** | **up** | **1.30E-09** | **2.55E-04** |
| ***FOXA1*** | **down** | **4.58E-11** | **4.42E-04** | ***ACTB*** | **up** | **7.33E-08** | **2.57E-04** |
| ***CDC37L1*** | **down** | **2.04E-08** | **4.82E-04** | ***UBE2S*** | **up** | **1.33E-05** | **2.62E-04** |
| ***FXYD1*** | **down** | **1.03E-06** | **4.83E-04** | ***SLC37A2*** | **up** | **1.26E-07** | **2.70E-04** |
| ***HSD17B6*** | **down** | **1.18E-06** | **4.86E-04** | ***COL3A1*** | **up** | **3.36E-08** | **2.95E-04** |
| ***KBTBD7*** | **down** | **2.69E-10** | **4.86E-04** | ***TPX2*** | **up** | **3.12E-04** | **3.13E-04** |
| ***KLHDC2*** | **down** | **5.00E-08** | **5.21E-04** | ***LDOC1*** | **up** | **2.89E-04** | **3.20E-04** |
| ***GLCE*** | **down** | **1.05E-05** | **5.32E-04** | ***RCN3*** | **up** | **1.40E-06** | **3.27E-04** |
| ***F10*** | **down** | **1.24E-05** | **5.34E-04** | ***FCRL5*** | **up** | **9.23E-12** | **3.28E-04** |
| ***PLS1*** | **down** | **1.41E-05** | **5.45E-04** | ***PRDM1*** | **up** | **3.07E-06** | **3.78E-04** |
| ***NRTN*** | **down** | **1.56E-08** | **5.87E-04** | ***EVI2A*** | **up** | **1.13E-08** | **3.80E-04** |
| ***SLAIN1*** | **down** | **2.24E-06** | **5.94E-04** | ***RNASEH2A*** | **up** | **2.27E-07** | **4.66E-04** |
| ***MME*** | **down** | **2.26E-08** | **6.34E-04** | ***LAX1*** | **up** | **3.23E-07** | **4.69E-04** |
| ***C6*** | **down** | **6.13E-12** | **6.57E-04** | ***OLFML2B*** | **up** | **6.67E-07** | **4.91E-04** |
| ***ZNF385B*** | **down** | **2.06E-14** | **7.30E-04** | ***MIAT*** | **up** | **1.02E-11** | **5.27E-04** |
| ***ALAS1*** | **down** | **8.36E-03** | **8.02E-04** | ***COL5A1*** | **up** | **5.28E-09** | **5.59E-04** |
| ***C4BPA*** | **down** | **1.23E-08** | **8.53E-04** | ***LRMP*** | **up** | **4.16E-06** | **5.95E-04** |
| ***FMO3*** | **down** | **6.12E-05** | **8.53E-04** | ***MS4A6E*** | **up** | **3.40E-04** | **5.98E-04** |
| ***ADAMTSL3*** | **down** | **1.33E-07** | **8.63E-04** | ***DOK3*** | **up** | **9.45E-09** | **7.20E-04** |
| ***DDC*** | **down** | **1.82E-04** | **9.01E-04** | ***KCNJ10*** | **up** | **6.48E-06** | **7.35E-04** |
| ***SLC25A18*** | **down** | **9.26E-05** | **9.07E-04** | ***GNA15*** | **up** | **9.47E-07** | **7.52E-04** |
| ***PLS3*** | **down** | **1.13E-07** | **1.06E-03** | ***RPS6KA1*** | **up** | **6.36E-09** | **7.54E-04** |
| ***LNX1*** | **down** | **2.85E-10** | **1.09E-03** | ***SIGLEC1*** | **up** | **3.79E-10** | **7.82E-04** |
| ***AADAC*** | **down** | **3.63E-03** | **1.11E-03** | ***TMC8*** | **up** | **4.70E-09** | **8.62E-04** |
| ***SCP2*** | **down** | **7.18E-12** | **1.12E-03** | ***CLEC11A*** | **up** | **8.57E-06** | **9.14E-04** |
| ***TMOD1*** | **down** | **2.26E-08** | **1.14E-03** | ***KIF23*** | **up** | **1.90E-06** | **9.16E-04** |
| ***FZD5*** | **down** | **4.88E-13** | **1.33E-03** | ***EBI3*** | **up** | **9.62E-06** | **9.24E-04** |
| ***C6orf211*** | **down** | **4.02E-07** | **1.41E-03** | ***AIM2*** | **up** | **2.85E-09** | **1.00E-03** |
| ***CTBS*** | **down** | **3.21E-11** | **1.42E-03** | ***C5AR1*** | **up** | **2.61E-08** | **1.13E-03** |
| ***CHSY3*** | **down** | **2.47E-06** | **1.50E-03** | ***WIPF1*** | **up** | **2.06E-11** | **1.17E-03** |
| ***ANKRD46*** | **down** | **1.09E-10** | **1.50E-03** | ***CDC20*** | **up** | **1.02E-07** | **1.20E-03** |
| ***ECHS1*** | **down** | **1.85E-04** | **1.55E-03** | ***DUSP4*** | **up** | **7.72E-11** | **1.32E-03** |
| ***ENPP1*** | **down** | **5.23E-08** | **1.68E-03** | ***COL1A1*** | **up** | **1.83E-08** | **1.34E-03** |
| ***FGGY*** | **down** | **1.01E-11** | **1.68E-03** | ***LY96*** | **up** | **1.72E-11** | **1.36E-03** |
| ***HRSP12*** | **down** | **2.05E-09** | **1.98E-03** | ***RCC2*** | **up** | **3.43E-10** | **1.39E-03** |
| ***CRYZ*** | **down** | **3.98E-05** | **2.05E-03** | ***RPL12*** | **up** | **3.05E-10** | **1.40E-03** |
| ***GRTP1*** | **down** | **2.76E-10** | **2.08E-03** | ***ARL4C*** | **up** | **2.29E-11** | **1.44E-03** |
| ***CPB2*** | **down** | **2.69E-08** | **2.17E-03** | ***TCIRG1*** | **up** | **1.83E-09** | **1.46E-03** |
| ***TMEM220*** | **down** | **9.92E-13** | **2.20E-03** | ***PFN1*** | **up** | **3.97E-06** | **1.62E-03** |
| ***AQP11*** | **down** | **3.47E-06** | **2.22E-03** | ***VCAN*** | **up** | **8.54E-09** | **1.72E-03** |
| ***C5*** | **down** | **6.19E-07** | **2.46E-03** | ***THBS1*** | **up** | **5.89E-04** | **1.74E-03** |
| ***UGP2*** | **down** | **9.07E-08** | **2.60E-03** | ***BACE2*** | **up** | **1.34E-07** | **1.76E-03** |
| ***FAM184A*** | **down** | **1.38E-08** | **2.66E-03** | ***PLEKHO1*** | **up** | **5.38E-10** | **1.90E-03** |
| ***MOCS2*** | **down** | **5.62E-11** | **2.81E-03** | ***RHOH*** | **up** | **4.78E-07** | **1.91E-03** |
| ***HOGA1*** | **down** | **4.74E-04** | **2.84E-03** | ***CSF2RB*** | **up** | **8.01E-05** | **1.91E-03** |
| ***MGST1*** | **down** | **1.55E-03** | **2.91E-03** | ***C12orf75*** | **up** | **9.64E-11** | **1.93E-03** |
| ***CAMK2N1*** | **down** | **2.82E-08** | **3.03E-03** | ***LPXN*** | **up** | **6.43E-08** | **1.97E-03** |
| ***SHROOM2*** | **down** | **6.90E-11** | **3.31E-03** | ***CXorf65*** | **up** | **5.34E-08** | **2.16E-03** |
| ***MMACHC*** | **down** | **1.41E-10** | **3.31E-03** | ***PBK*** | **up** | **3.25E-03** | **2.43E-03** |
| ***MID1IP1*** | **down** | **1.06E-09** | **3.37E-03** | ***COMP*** | **up** | **3.94E-09** | **2.59E-03** |
| ***FOLH1B*** | **down** | **6.73E-11** | **3.54E-03** | ***THBS2*** | **up** | **3.59E-08** | **2.67E-03** |
| ***SLC25A43*** | **down** | **1.75E-05** | **3.55E-03** | ***HELLS*** | **up** | **8.80E-09** | **2.67E-03** |
| ***MAP7*** | **down** | **2.86E-11** | **3.62E-03** | ***MXRA8*** | **up** | **6.52E-08** | **2.78E-03** |
| ***CENPV*** | **down** | **1.21E-05** | **3.64E-03** | ***LSM7*** | **up** | **2.03E-08** | **2.91E-03** |
| ***SLC6A12*** | **down** | **1.43E-07** | **3.68E-03** | ***CD27*** | **up** | **1.34E-06** | **3.13E-03** |
| ***DCXR*** | **down** | **5.23E-03** | **3.73E-03** | ***KIAA0125*** | **up** | **6.67E-06** | **3.22E-03** |
| ***ATG2B*** | **down** | **1.85E-11** | **3.78E-03** | ***UBE2T*** | **up** | **1.65E-09** | **3.36E-03** |
| ***ACVR1C*** | **down** | **4.03E-11** | **3.98E-03** | ***IL2RA*** | **up** | **3.50E-08** | **3.57E-03** |
| ***AOX1*** | **down** | **3.32E-07** | **4.04E-03** | ***SRPX2*** | **up** | **1.27E-08** | **4.02E-03** |
| ***MXI1*** | **down** | **3.69E-06** | **4.15E-03** | ***ARHGAP9*** | **up** | **1.82E-11** | **4.07E-03** |
| ***MAP2K1*** | **down** | **1.32E-09** | **4.20E-03** | ***CXCL14*** | **up** | **2.73E-07** | **4.10E-03** |
| ***MAOA*** | **down** | **3.54E-06** | **4.59E-03** | ***COL4A1*** | **up** | **3.68E-11** | **4.11E-03** |
| ***TMEM41B*** | **down** | **6.16E-13** | **4.65E-03** | ***CCL19*** | **up** | **7.19E-04** | **4.57E-03** |
| ***SLC19A3*** | **down** | **7.59E-10** | **4.82E-03** | ***UCA1*** | **up** | **1.76E-04** | **4.71E-03** |
| ***SOCS6*** | **down** | **3.14E-08** | **5.25E-03** | ***WISP1*** | **up** | **1.76E-08** | **4.75E-03** |
| ***HMGCR*** | **down** | **6.34E-07** | **5.33E-03** | ***TIMD4*** | **up** | **5.83E-07** | **4.79E-03** |
| ***KBTBD6*** | **down** | **6.62E-07** | **5.41E-03** | ***RRM2*** | **up** | **2.54E-07** | **4.82E-03** |
| ***ASL*** | **down** | **1.02E-04** | **5.44E-03** | ***MMP1*** | **up** | **2.98E-04** | **5.11E-03** |
| ***TOM1L1*** | **down** | **5.81E-09** | **5.45E-03** | ***MICB*** | **up** | **1.18E-08** | **5.20E-03** |
| ***ALS2*** | **down** | **9.22E-12** | **5.73E-03** | ***TNFSF13B*** | **up** | **2.06E-09** | **5.23E-03** |
| ***GSTZ1*** | **down** | **6.55E-04** | **5.80E-03** | ***EFCAB4A*** | **up** | **1.84E-07** | **5.39E-03** |
| ***CEBPA*** | **down** | **1.37E-06** | **6.12E-03** | ***UHRF1*** | **up** | **6.04E-07** | **5.58E-03** |
| ***RMND5A*** | **down** | **5.34E-10** | **6.14E-03** | ***P2RY13*** | **up** | **8.60E-07** | **6.12E-03** |
| ***SAMD5*** | **down** | **4.20E-12** | **6.14E-03** | ***GCNT3*** | **up** | **1.81E-05** | **6.13E-03** |
| ***AJUBA*** | **down** | **3.63E-07** | **6.15E-03** | ***KRT7*** | **up** | **3.19E-11** | **6.49E-03** |
| ***2-Mar*** | **down** | **7.62E-14** | **6.24E-03** | ***ADAP2*** | **up** | **2.39E-07** | **6.78E-03** |
| ***ALDH2*** | **down** | **4.50E-17** | **6.37E-03** | ***FANCI*** | **up** | **2.56E-08** | **6.92E-03** |
| ***PCGF6*** | **down** | **2.25E-09** | **6.44E-03** | ***SLC45A4*** | **up** | **9.53E-07** | **7.01E-03** |
| ***EPT1*** | **down** | **2.16E-07** | **6.64E-03** | ***EIF3G*** | **up** | **5.12E-09** | **7.01E-03** |
| ***PDSS2*** | **down** | **5.84E-10** | **6.70E-03** | ***SECTM1*** | **up** | **3.48E-09** | **7.26E-03** |
| ***TSPAN6*** | **down** | **3.32E-07** | **6.75E-03** | ***NINJ1*** | **up** | **8.91E-08** | **7.47E-03** |
| ***ECI2*** | **down** | **1.07E-04** | **6.90E-03** | ***GPX8*** | **up** | **1.73E-05** | **8.12E-03** |
| ***RNASE4*** | **down** | **7.05E-07** | **7.01E-03** | ***HMOX1*** | **up** | **5.17E-10** | **8.26E-03** |
| ***TCEA3*** | **down** | **1.93E-06** | **7.19E-03** | ***BUB1B*** | **up** | **4.36E-08** | **9.39E-03** |
| ***TMEM64*** | **down** | **5.21E-07** | **7.30E-03** | ***TBC1D10C*** | **up** | **2.84E-12** | **9.48E-03** |
| ***KLB*** | **down** | **4.67E-08** | **7.31E-03** | ***CENPF*** | **up** | **5.11E-06** | **9.75E-03** |
| ***SYT17*** | **down** | **2.61E-10** | **7.38E-03** | ***COL10A1*** | **up** | **7.68E-07** | **1.00E-02** |
| ***IYD*** | **down** | **2.34E-07** | **7.49E-03** | ***NPL*** | **up** | **1.32E-10** | **1.01E-02** |
| ***HMGCL*** | **down** | **2.00E-07** | **7.51E-03** | ***SPDYE2*** | **up** | **2.46E-05** | **1.06E-02** |
| ***GPAM*** | **down** | **3.20E-06** | **7.52E-03** | ***COL5A2*** | **up** | **1.61E-07** | **1.18E-02** |
| ***ISOC1*** | **down** | **3.65E-07** | **7.54E-03** | ***EID3*** | **up** | **7.01E-06** | **1.25E-02** |
| ***GCHFR*** | **down** | **9.90E-06** | **7.63E-03** | ***FGD2*** | **up** | **5.70E-11** | **1.25E-02** |
| ***PZP*** | **down** | **3.39E-08** | **7.82E-03** | ***HMMR*** | **up** | **2.96E-05** | **1.31E-02** |
| ***ADK*** | **down** | **1.31E-07** | **8.36E-03** | ***FLJ20021*** | **up** | **1.48E-06** | **1.39E-02** |
| ***GAS2*** | **down** | **4.20E-07** | **8.67E-03** | ***CTSA*** | **up** | **3.71E-10** | **1.42E-02** |
| ***BCKDHB*** | **down** | **7.69E-11** | **9.02E-03** | ***ZAP70*** | **up** | **3.33E-12** | **1.46E-02** |
| ***ACACB*** | **down** | **3.10E-14** | **9.65E-03** | ***ITGA2*** | **up** | **2.23E-10** | **1.49E-02** |
| ***HHEX*** | **down** | **1.94E-10** | **9.87E-03** | ***DBN1*** | **up** | **2.27E-09** | **1.51E-02** |
| ***LRIF1*** | **down** | **4.01E-06** | **9.92E-03** | ***HLA-C*** | **up** | **7.38E-11** | **1.63E-02** |
| ***LACTB2*** | **down** | **1.67E-04** | **1.04E-02** | ***RAB38*** | **up** | **2.85E-07** | **1.63E-02** |
| ***ABHD15*** | **down** | **7.68E-05** | **1.06E-02** | ***LILRA3*** | **up** | **1.55E-06** | **1.66E-02** |
| ***GFPT1*** | **down** | **4.54E-08** | **1.10E-02** | ***SIGLEC9*** | **up** | **3.25E-09** | **1.67E-02** |
| ***STRADB*** | **down** | **4.71E-09** | **1.16E-02** | ***SELM*** | **up** | **5.38E-08** | **1.71E-02** |
| ***ADIPOR2*** | **down** | **2.61E-07** | **1.17E-02** | ***PNMAL1*** | **up** | **1.68E-03** | **1.73E-02** |
| ***C11orf54*** | **down** | **1.65E-09** | **1.21E-02** | ***IFI27L2*** | **up** | **1.20E-06** | **1.74E-02** |
| ***SLC27A2*** | **down** | **1.44E-03** | **1.29E-02** | ***PRF1*** | **up** | **1.06E-11** | **1.74E-02** |
| ***ZFAND1*** | **down** | **3.68E-08** | **1.30E-02** | ***GPX7*** | **up** | **4.36E-05** | **1.81E-02** |
| ***ANO5*** | **down** | **2.60E-09** | **1.32E-02** | ***CEP55*** | **up** | **1.56E-05** | **1.82E-02** |
| ***ERRFI1*** | **down** | **6.36E-04** | **1.32E-02** | ***FOXQ1*** | **up** | **1.25E-03** | **1.85E-02** |
| ***MTM1*** | **down** | **2.90E-07** | **1.36E-02** | ***CKS2*** | **up** | **1.20E-05** | **1.86E-02** |
| ***AGL*** | **down** | **1.87E-10** | **1.40E-02** | ***DENND1C*** | **up** | **9.62E-09** | **1.88E-02** |
| ***GPR146*** | **down** | **1.59E-05** | **1.40E-02** | ***SLAMF8*** | **up** | **5.47E-11** | **1.90E-02** |
| ***LOC286161*** | **down** | **3.09E-09** | **1.40E-02** | ***C1orf54*** | **up** | **1.08E-10** | **2.07E-02** |
| ***TPD52L1*** | **down** | **4.57E-04** | **1.40E-02** | ***KIF4A*** | **up** | **1.75E-05** | **2.12E-02** |
| ***DIRAS3*** | **down** | **7.46E-08** | **1.44E-02** | ***SLC26A10*** | **up** | **1.53E-04** | **2.22E-02** |
| ***GPR133*** | **down** | **7.87E-04** | **1.44E-02** | ***C16orf54*** | **up** | **2.11E-09** | **2.32E-02** |
| ***CYP2J2*** | **down** | **1.53E-05** | **1.44E-02** | ***FBLN2*** | **up** | **2.35E-04** | **2.42E-02** |
| ***TMEM14A*** | **down** | **7.74E-05** | **1.45E-02** | ***CR1*** | **up** | **3.27E-08** | **2.47E-02** |
| ***AK3*** | **down** | **7.50E-07** | **1.46E-02** | ***CENPW*** | **up** | **1.67E-07** | **2.50E-02** |
| ***EBPL*** | **down** | **8.38E-08** | **1.49E-02** | ***ATG16L2*** | **up** | **4.48E-11** | **2.59E-02** |
| ***DENND4A*** | **down** | **5.16E-09** | **1.51E-02** | ***PLTP*** | **up** | **1.60E-07** | **2.62E-02** |
| ***FAAH2*** | **down** | **1.48E-08** | **1.54E-02** | ***ASPHD2*** | **up** | **3.77E-10** | **2.70E-02** |
| ***ST6GAL1*** | **down** | **1.91E-15** | **1.56E-02** | ***SIRPB2*** | **up** | **1.78E-08** | **2.71E-02** |
| ***ARFGAP3*** | **down** | **3.93E-08** | **1.56E-02** | ***HES4*** | **up** | **1.19E-09** | **2.71E-02** |
| ***MCC*** | **down** | **2.60E-11** | **1.58E-02** | ***ANGPTL4*** | **up** | **2.75E-03** | **2.74E-02** |
| ***MYOM2*** | **down** | **1.97E-07** | **1.64E-02** | ***CTSE*** | **up** | **3.64E-11** | **2.75E-02** |
| ***SLC22A3*** | **down** | **1.10E-04** | **1.66E-02** | ***DOK2*** | **up** | **9.30E-09** | **2.79E-02** |
| ***MMRN1*** | **down** | **3.04E-04** | **1.66E-02** | ***TMEM200A*** | **up** | **1.59E-04** | **2.84E-02** |
| ***RBMXL1*** | **down** | **4.46E-06** | **1.67E-02** | ***HLA-G*** | **up** | **3.14E-09** | **2.88E-02** |
| ***ACADL*** | **down** | **1.79E-09** | **1.67E-02** | ***COL16A1*** | **up** | **1.73E-06** | **2.98E-02** |
| ***ATP7B*** | **down** | **3.45E-08** | **1.69E-02** | ***NR1H3*** | **up** | **2.16E-07** | **3.06E-02** |
| ***PTPN3*** | **down** | **1.48E-10** | **1.69E-02** | ***HAVCR2*** | **up** | **3.89E-10** | **3.16E-02** |
| ***LCAT*** | **down** | **4.27E-11** | **1.73E-02** | ***SPC25*** | **up** | **2.34E-03** | **3.16E-02** |
| ***OCLN*** | **down** | **2.47E-08** | **1.73E-02** | ***ANKRD36BP2*** | **up** | **1.98E-12** | **3.27E-02** |
| ***C6orf123*** | **down** | **1.04E-07** | **1.75E-02** | ***FYB*** | **up** | **1.00E-08** | **3.31E-02** |
| ***ADRB2*** | **down** | **2.47E-06** | **1.85E-02** | ***PIM2*** | **up** | **1.87E-11** | **3.61E-02** |
| ***PCOLCE2*** | **down** | **1.18E-12** | **1.92E-02** | ***GINS1*** | **up** | **8.68E-06** | **3.66E-02** |
| ***FMO5*** | **down** | **6.46E-08** | **1.93E-02** | ***DDB2*** | **up** | **3.27E-05** | **3.68E-02** |
| ***RNF128*** | **down** | **1.04E-04** | **1.95E-02** | ***NCK2*** | **up** | **1.95E-05** | **3.70E-02** |
| ***GFM1*** | **down** | **4.86E-09** | **1.98E-02** | ***GBP5*** | **up** | **8.31E-11** | **3.74E-02** |
| ***DHCR24*** | **down** | **1.28E-06** | **2.04E-02** | ***TMEM106C*** | **up** | **5.01E-10** | **3.78E-02** |
| ***LYRM5*** | **down** | **8.26E-10** | **2.04E-02** | ***GZMH*** | **up** | **8.92E-13** | **3.86E-02** |
| ***ADH1A*** | **down** | **1.05E-04** | **2.12E-02** | ***CCR5*** | **up** | **3.22E-10** | **3.98E-02** |
| ***VNN3*** | **down** | **3.65E-10** | **2.14E-02** | ***FJX1*** | **up** | **5.03E-07** | **3.99E-02** |
| ***IRF6*** | **down** | **4.29E-12** | **2.16E-02** | ***IL32*** | **up** | **9.79E-03** | **4.05E-02** |
| ***SPPL2A*** | **down** | **6.85E-10** | **2.24E-02** | ***RASGRP1*** | **up** | **7.17E-09** | **4.15E-02** |
| ***GPN3*** | **down** | **9.96E-07** | **2.29E-02** | ***FHL2*** | **up** | **2.73E-06** | **4.15E-02** |
| ***DTX4*** | **down** | **1.32E-08** | **2.33E-02** | ***SPP1*** | **up** | **4.34E-07** | **4.18E-02** |
| ***TMEM45A*** | **down** | **2.08E-09** | **2.34E-02** | ***BLM*** | **up** | **4.74E-08** | **4.32E-02** |
| ***HADH*** | **down** | **2.09E-06** | **2.42E-02** | ***METRNL*** | **up** | **2.36E-09** | **4.49E-02** |
| ***DNAJB9*** | **down** | **1.10E-09** | **2.44E-02** | ***DEFA4*** | **up** | **8.34E-03** | **4.49E-02** |
| ***FDX1*** | **down** | **7.15E-11** | **2.44E-02** | ***ITGAM*** | **up** | **1.04E-06** | **4.62E-02** |
| ***FRK*** | **down** | **8.69E-08** | **2.45E-02** | ***NUF2*** | **up** | **6.00E-08** | **4.74E-02** |
| ***PEX3*** | **down** | **2.34E-09** | **2.52E-02** | ***KIF11*** | **up** | **1.47E-08** | **4.74E-02** |
| ***SYBU*** | **down** | **1.53E-09** | **2.52E-02** | ***FOLR2*** | **up** | **1.75E-11** | **4.97E-02** |
| ***TMEM192*** | **down** | **5.77E-10** | **2.59E-02** |  |  |  |  |
| ***AGBL2*** | **down** | **1.47E-08** | **2.60E-02** |  |  |  |  |
| ***THNSL1*** | **down** | **3.04E-05** | **2.61E-02** |  |  |  |  |
| ***AKTIP*** | **down** | **1.75E-08** | **2.68E-02** |  |  |  |  |
| ***ALDH5A1*** | **down** | **2.08E-10** | **2.85E-02** |  |  |  |  |
| ***SLITRK3*** | **down** | **1.31E-04** | **2.86E-02** |  |  |  |  |
| ***HOOK1*** | **down** | **1.09E-09** | **2.86E-02** |  |  |  |  |
| ***HLF*** | **down** | **1.91E-05** | **3.05E-02** |  |  |  |  |
| ***CCT6B*** | **down** | **1.79E-06** | **3.15E-02** |  |  |  |  |
| ***GOLT1A*** | **down** | **1.24E-02** | **3.19E-02** |  |  |  |  |
| ***KLF15*** | **down** | **1.69E-11** | **3.21E-02** |  |  |  |  |
| ***COQ10A*** | **down** | **4.66E-16** | **3.30E-02** |  |  |  |  |
| ***ECHDC3*** | **down** | **1.63E-03** | **3.35E-02** |  |  |  |  |
| ***CYP2C8*** | **down** | **1.95E-05** | **3.48E-02** |  |  |  |  |
| ***RTP3*** | **down** | **4.37E-05** | **3.53E-02** |  |  |  |  |
| ***TMEM56*** | **down** | **2.95E-08** | **3.55E-02** |  |  |  |  |
| ***PPP1R3C*** | **down** | **5.71E-13** | **3.57E-02** |  |  |  |  |
| ***AUH*** | **down** | **9.49E-07** | **3.63E-02** |  |  |  |  |
| ***TIGD2*** | **down** | **7.48E-12** | **3.75E-02** |  |  |  |  |
| ***CERS2*** | **down** | **2.17E-08** | **3.78E-02** |  |  |  |  |
| ***TMEM176B*** | **down** | **2.43E-09** | **3.79E-02** |  |  |  |  |
| ***PTPRF*** | **down** | **1.03E-06** | **3.83E-02** |  |  |  |  |
| ***SLCO4C1*** | **down** | **8.83E-14** | **3.93E-02** |  |  |  |  |
| ***LINC00261*** | **down** | **5.98E-05** | **3.98E-02** |  |  |  |  |
| ***CPS1*** | **down** | **2.14E-02** | **4.20E-02** |  |  |  |  |
| ***CDO1*** | **down** | **6.52E-07** | **4.35E-02** |  |  |  |  |
| ***APOH*** | **down** | **1.88E-05** | **4.75E-02** |  |  |  |  |
| ***DNAH5*** | **down** | **1.83E-05** | **4.75E-02** |  |  |  |  |
| ***PHYH*** | **down** | **1.88E-06** | **4.76E-02** |  |  |  |  |
| ***TMED6*** | **down** | **9.94E-07** | **4.79E-02** |  |  |  |  |
| ***MTHFD1*** | **down** | **4.27E-05** | **4.79E-02** |  |  |  |  |
| ***GPT2*** | **down** | **1.81E-02** | **4.85E-02** |  |  |  |  |
| ***C1D*** | **down** | **1.91E-07** | **4.91E-02** |  |  |  |  |
| ***QDPR*** | **down** | **1.03E-06** | **4.95E-02** |  |  |  |  |
| ***IL17RB*** | **down** | **5.10E-05** | **4.99E-02** |  |  |  |  |
