# Supplementary material for: Exploration and verification of COVID-19-related hub genes in liver physiological and pathological regeneration
Source: Front Bioeng Biotechnol. 2023 Feb 23;11:1135997. doi: 10.3389/fbioe.2023.1135997 (PMC9997844; doi:10.3389/fbioe.2023.1135997)
Supplement: Supplementary file 2 [file DataSheet1.docx]

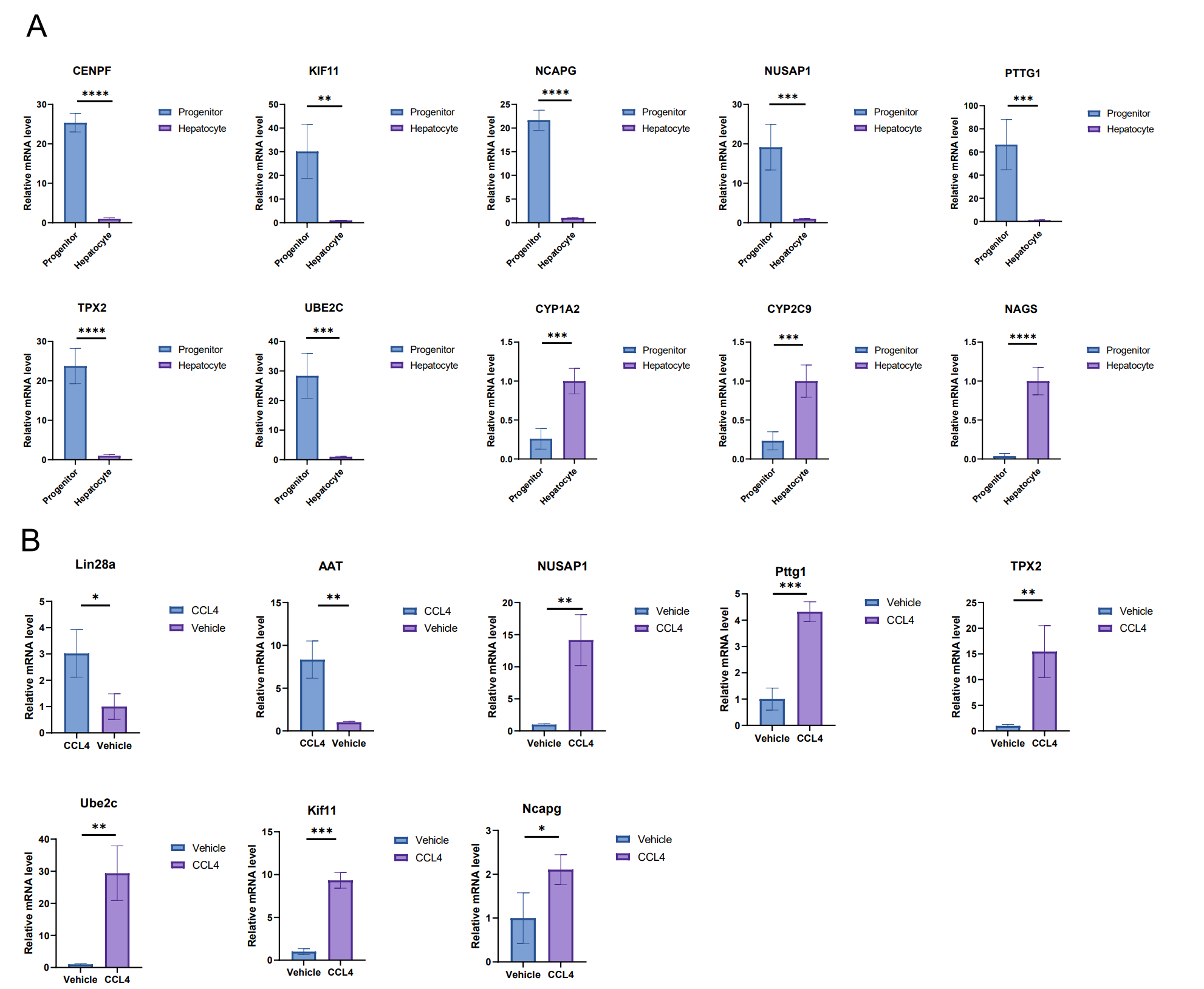


**S1.** (A) The relative expression of hub genes, progenitor genes and liver functional genes between hepatocytes and progenitors. N=4 (B) The relative expression of hub genes, progenitor genes and functional genes between ALF and vehicle mice. N=3. *indicates significant difference at p < 0.05, ** indicates significant difference at p < 0.01, *** indicates significant difference at p < 0.001, **** indicates significant difference at P <0.0001
